# Food safety in Vietnam: where we are at and what we can learn from international experiences

**DOI:** 10.1186/s40249-017-0249-7

**Published:** 2017-02-16

**Authors:** Hung Nguyen-Viet, Tran Thi Tuyet-Hanh, Fred Unger, Sinh Dang-Xuan, Delia Grace

**Affiliations:** 1 International Livestock Research Institute (ILRI), Room 301-302, B1 Building, Van Phuc Diplomatic Compound, 298 Kim Ma Street, Ba Dinh District, Hanoi, Vietnam; 2 Centre for Public Health and Ecosystem Research, Hanoi University of Public Health, 1A Duc Thang Road, North Tu Liem, Hanoi, Vietnam

**Keywords:** Food safety, Risk communication, Health risk, Economic impact, Vietnam

## Abstract

Food-borne diseases are attracting a lot of attention in Vietnam as a result of repeated episodes of adulterated and unsafe food. In this paper, we provide some perspectives on food safety in Vietnam from the point of view of an international research institution working on food safety with partners in the country. We argue that one of the key issues of food safety in Vietnam is that certain food value chain stakeholders lack ethics, which leads to the production and trading of unsafe foods in order to make profits irrespective of adverse health effects on consumers. In turn, the shortfall in ethical behaviours around food can be attributed to a lack of incentives or motivating factors.

Although food safety causes panic in the population, it is unclear how much contaminated food contributes to the burden of food-borne diseases and food poisonings in Vietnam. However, globally, the biggest health problem associated with food are infections from consuming food contaminated with viruses, bacteria or parasites. A major food safety challenge is the inappropriate way of communicating food risks to the public. Another key constraint is the inherent difficulty in managing food in wet markets and from smallholder production. On the other hand, local foods, and local food production and processing are an important cultural asset as well as being essential to food safety, and these aspects can be put at risk if food safety concerns motivate consumers to purchase more imported foods.

In this paper, we also discuss good experiences in food safety management from other countries and draw lessons learnt for Vietnam on how to better deal with the current food safety situation.

**Electronic supplementary material:**

The online version of this article (doi:10.1186/s40249-017-0249-7) contains supplementary material, which is available to authorized users.

## Multilingual abstract

Please see Additional file 1 for translations of abstract into six official working languages of the United Nations.

## Background

Food-borne diseases and food poisonings are attracting a lot of attention in Vietnam due to repeated episodes of adulterated and unsafe food practices receiving widespread media attention. For example, it was recently reported that nine tonnes of salbutamol were legally imported for medical purposes in 2015, but only 10 kg were actually needed yearly for human use – the rest was likely used for livestock growth promotion [1]. And this is just the latest in a long line of food scares, which include: pesticide residues in vegetables; antibiotic and banned veterinary residues in meat; urea used for fish conservation; salvaging and consuming of spoiled animal-sourced food; and high levels of microbial contamination in meat.

The Vietnamese media gives a lot of attention to food safety issues when famous people pass away at a young age from cancers, asking whether there is something wrong with our food. The countries’ top leaders, too, have discussed food safety issues at meetings of the national assembly. Indeed, a minister was criticised for remarking, *“the majority of foods in Vietnam are safe, but people just do not know this”*, and had to publically apologise for this misleading statement, something rare in Vietnam and a sign of huge public concern. On 1^st^ April 2016, an official programme was launched on national television, entitled *Say no to contaminated foods*, which is being broadcasted daily during two primetime slots – 7:30 am and 8:30 pm – on Vietnam television (VTV) 1, VTV8 and VTV9, from Monday to Friday [2].

In this paper, we wish to present a perspective on food safety in Vietnam in the context of an international research institution working on food safety with partners in Vietnam and internationally. As we work more with animal-sourced food, we place more focus on these and discuss vegetables only to some extent.

## Main text

### Ethics: profiting despite of adverse health outcomes for consumers

The health of the public is put at risk when stakeholders along the food chain do not follow good practices of producing, processing, conserving, transporting and selling food. This leads to contamination of animal feeds by banned chemicals, sale of spoiled foods, using chemicals to make fake beef from lean pork meat etc., and other unethical behaviours. Farmers are reported to produce safe or safer foods for their own consumption, while selling unsafe foods to the public. There is little trust among stakeholders, but this is not the fault of individual farmers and traders. Rather, it is the predicament of a food system that has developed in a way that provides little rewards for those who practice good safety, but high rewards for those who carry out bad and unsafe practices [3]. This high prevalence of poor practices was also very common in Europe and America during times of rapid development, and it is a problem that can be overcome [4].

A common response to the concerns over food safety is an attempt to strengthen regulations, and ramp up inspections and punishments. This has also been seen in Vietnam. For example, during the week of 20^th^ May 2016, 80 pigs from Dong Nai Province were found to have been contaminated with salbutamol, with the farm owner being fined VND 25 million (approximately US$ 1 100) and all the contaminated pigs being disposed of [5]. The use of banned substances in animal husbandry will now face harsher punishment as the new criminal code took effect on 1^st^ July 2016. Yet, the experiences of developed countries, which now have relatively safe food, is that command-and-control approaches to food safety, which rely mainly on inspection and punishment, are less effective than approaches in which stakeholders are empowered and encouraged to self-regulate, motivated by the realisation that this is more profitable in the long term. With these approaches, the emphasis moves away from testing the safety of end products to assuring that the process of food production remains within safe limits at all times.

### Burden of diseases caused by unsafe foods

The first step to rational management of food safety practices is forming an understanding of the health burden of unsafe food and where it is coming from. Vietnam had 125 000 new cases of cancers reported in 2012 (140 cases per 100 000 population); this is predicted to increase to 190 000 new cases by 2020, when 75 000 deaths will result from cancers per year (84 deaths per 100 000 population) [6]. There is a very common belief that eating foods contaminated with pesticides or other chemicals is an important cause of cancer. However, the proportion of cancers caused by contaminated food in Vietnam is unknown. Generally, there is far more concern about the carcinogenic impact of food than the evidence to support this. For example, three important pesticides are often implicated as carcinogenic: diazinon, malathion and glycophosphate. Recently, however, an expert committee of the World Health Organisation (WHO) and the Food and Agriculture Organization concluded that these pesticides were unlikely to pose a carcinogenic risk through dietary exposure [7]. A major reason for more cancer diagnoses is that people are living longer and diagnoses are becoming more accurate. Undoubtedly, some cancers are associated with diet, but risky behaviours (e.g. smoking, alcohol abuse) and environmental factors are also important, especially in places where environmental quality is greatly degraded. However, there is much that is unknown about the long-term effects of chemicals in food.

Surprisingly, the greatest health problem associated with food are infections that result from food contaminated with bacteria, viruses and parasites. The first-ever report of the global burden of food-borne diseases recently released by the WHO shows that the burden from food-borne diseases is at the level of the ‘big three’ (HIV/AIDS, tuberculosis and malaria) [8]. The Western Pacific region where Vietnam is part of ranks second in the world in terms of food-borne diseases. In this region, at least 50 000 people die from food contamination and more than 125 million people become ill from food each year out of the estimated 1.5 billion inhabitants – meaning that eight in every 100 people fall ill [8].

### Issues of risk communication

Risk communication regarding food safety is often poor, which makes consumers even more frightened about the foods they purchase. For example, in one incident in China, meat was contaminated with phosphorescent bacteria, which caused it to glow in the dark. Authorities informed the public that the meat was safe to eat. Although this was likely true, it just made people more scared and angry at the authorities [9]. However, when pork was found to be contaminated with dioxins in Ireland, authorities withdrew all pork even though the European Food Safety Authority and the Irish authorities confirmed there was no risk to human health from the dioxins. Later, surveys revealed that most people found the way in which the authorities managed the crisis was ‘adequate’ or ‘very efficient’, and trust remained high in the Irish food system [10]. In fact, in Europe, public health practitioners generally practice the precautionary and would advise that the procedure followed by the Irish precautionary principle authorities was correct and appropriate [11].

To communicate risk effectively, it is important to understand the psychology of risk perception. People encounter information from different sources about chemicals detected in food. Consumers normally do not think about risk in the same way that risk assessors understand risk. People filter information through a variety of lenses that affect their perceptions of the risks and what they can actually do to minimise them. For example, as mentioned earlier, biological hazards in some foods may cause more sickness and death than chemical hazards, but consumers are usually more worried about chemical hazards [8, 9].

Risk perception is complex and driven only partly by factual evidence. Food technologies often involve ‘fear factors’ that make them seem more worrisome than other risks – for example, eating pesticide-contaminated vegetables is (incorrectly) perceived as being more risky than riding a motorbike. Fear factors include distrust of large companies, dislike of ‘unnatural’ processes and uncertainty over unfamiliar dangers. Risks that go along with benefits to the consumer (e.g. convenience food) are often found to be more acceptable than risks where benefits are accrued by the food industry. We believe that people tend to worry more about risks caused by factors over which they feel they have no control, while being much less concerned about factors linked to their own behaviours. People are not very good at seeking out better evidence about risks and are more influenced by bad news than good news.

The marked difference in how experts and the public view food safety risks has real consequences: opportunities are lost and scarce resources are spent managing minor problems, while the major issues go to the back of the queue. Effective regulation of risk, hence, poses a singular challenge to democracy, and our natural tendencies to misperceive risk need to be countered by better evidence, not only on the risks themselves, but also on the psychology of risk perception.

Communication that builds on empirical evidence of, and interactive exchanges about, consumer understanding, as well as on food risks and benefits can help consumers make informed decisions [12]. The risk assessment of chemical, biological and physical hazards in foods is crucial for providing scientific information on the actual risk and informing official risk communication activities. However, currently, risk communication on food safety issues has not been integrated into the recommended risk-based food safety management system in Vietnam, as specified in the Food Safety Law 2010 [13].

### Economic implications and the role of smallholders in food safety

Vietnam is a member of several free-trade agreements, in particular the Trans-Pacific Partnership, so the likelihood of increasing imports of affordable and quality foods from other countries is real. This presents challenges to domestic food production in Vietnam, especially to the smallholders. For example, Australian beef is cheaper than Vietnamese beef, US chicken is cheaper than Vietnamese chicken and European countries have started negotiating the export of pork to Vietnam. This put livestock smallholder in a difficult position of being less competitive. However, smallholders are clearly key to food security and agricultural development as they produce 90% of vegetables and 65% of pork in the domestic market [14]. Women also have an important role in food production, as they make up the majority of meat sellers, so food production also has important benefits for equity. In the long term, we expect large-scale and industrial production and retail to become more common, however, small-scale production and informal retail will still last for decades. For example, pig sector modelling predicted that smallholder pig production will continue for the next 15–20 years [14]. Hence, the lack of food safety in the country’s food chain could be a risk to food security and the agricultural sector if it motivates people to switch to imported food that is both cheaper and safer at the expense of domestic production.

### Food safety solutions

So what are some of the solutions to the food safety problem in Vietnam? The experiences of different countries can provide lessons for improving food safety in the country.

Several countries have succeeded in reducing food-borne diseases over relatively short periods. The UK reversed an epidemic of *Salmonella* through legislation, food safety advice and an industry-led vaccination scheme covering broiler-breeder and laying poultry flocks [15]. In Iceland, measures at the production, retail and household levels, such as public education, enhanced on-farm biological security measures and carcass freezing, resulted in *Campylobacter* declines of more than 70% in broiler flocks and in humans [16]. Denmark reduced *Salmonella* by up to 95% in eggs, poultry and pork by monitoring herds and flocks, eliminating infected animals and differential processing depending on *Salmonella* contamination status. This resulted in savings of US$ 25.5 million [17].

In all three of these success stories, control was incorporated into the value chain, with an emphasis on reducing disease in the animal reservoir rather than in the retail product. However, these control approaches are mainly applicable to industrialised countries with modern intensive farming systems and good enforcement capacity, and may not be directly applied in Vietnam, where the majority of foods are produced by smallholders and food safety regulation enforcement is quite weak.

Yet, during the past several decades, there have been a number of initiatives to improve the safety of fresh vegetables and meat in Vietnam, with varying successes and challenges. A major government approach has been the development of a standards scheme based on Good Agricultural Practices (GAP). However, this scheme involves high costs and demands a lot of effort from farmers, making it less suitable for some. In Vietnam, GAP have been introduced for crop farming, livestock and aquaculture, but uptake is less than 1% [18]. Moreover, while studies have found that smallholders participating in export GAP are improving their livelihoods and producing food of acceptable quality [19], there is little evidence to suggest that participation in domestic GAP is profitable or makes food safer. For example, in Thailand, farmers who follow the public GAP do not have better pesticide use or outcomes than those who do not [20]. In fact, qualitative evidence points to poor implementation of farm auditing related to a programme expansion that was too rapid, a lack of understanding among farmers about the logic of the control points in the standard, and a lack of alternatives given to farmers to manage their pest problems. The author argued that by focusing on the testing of farm produce for pesticide residues, the public GAP programme is paying too much attention to the consequences rather than the root cause of the problem, and this needs to be balanced.

Upgrading value chains and certifying safety have also received governmental and project support. During the avian influenza outbreak, certified birds were available in projects that supported influenza controls. However, 72% of consumers never purchased certified birds and while nearly 40% of respondents regularly buy chickens that have governmental certification stamps, they do not see these as a credible certification [21]. Lack of trust in certification, inconvenience and lack of interest were key reasons for not purchasing safety-branded chicken.

Likewise, experiences in vegetable chain in Vietnam are challenged by a lack of trust in vegetable certification and the premium associated with branded vegetables. After more than 10 years of major efforts and investments by state authorities and market stakeholders, the safe vegetable production and distribution system has not yet been able to take a significant share of the vegetable market and gain widespread consumer trust [22]. Vegetables certified as safe are less than 10% of the total sold, and it is our opinion that there is weak evidence that certified products are actually safer than traditionally produced and marketed vegetables.

Governmental officials often see modernising retail as the way forward for improving food safety. However, this is challenged by high costs, consumer preference for warm fresh meat, resistance from retailers [23], as well as the inability to show improvements in safety [24].

The International Livestock Research Institute (ILRI, https://www.ilri.org) has been developing market-based approaches to improving food safety in informal markets. This was first developed for the informal milk sector in Kenya and has been subsequently extended to the dairy sector in Assam in India and Tanzania, and meat retail in a large Nigerian metropolis [25]. The central idea is light-touch interventions that are sustainable and scalable, changing practice through capacity building for food safety actors such as farmers, slaughtering workers, butchers and incentives, and providing an enabling policy environment. The approach has been positively reviewed by the Institute of Development Studies as an example of making markets work for the poor [26]. For example, an ILRI project trained butchers from butcher associations in Nigeria to improve their hygiene practices, taught them about which behaviours created the greatest risks and listened as the butchers discussed their own experiences. This led to the development of a set of feasible best practices. We compared butchers’ practices before and after the workshop with the practices of non-participating butchers in order to assess whether butchers’ associations had disseminated the best practices to non-participants. Gender had a major influence on food safety outcomes as they play different role in food safety risk management. For example women are mainly responsible for buying and preparing food whereas men are involved more in food production and slaughtering. Training appeared to improve certain hygiene practices, with 85% of butchers reporting using disinfectant after the training, compared to 48% before. Furthermore, the butchers’ associations seemed to have diffused these behaviours among their members; training attendees and non-attendees were equally likely to report using many key hygiene practices [25].

Different contexts require different approaches and reveal different incentives. In all cases, capacity building, incentives for behaviour change and enabling policy were key to scale and sustainability. In Kenya, a major incentive for behaviour change was obtaining a certificate that provided protection from harassment by authorities; in Assam, it was the inclusion – for the first time – of dairy traders’ associations in dialogue with the government. In both countries, business performance improved as a result of the training. Peer-reviewed evaluations of the work in Kenya and Assam have shown the promise of this approach [27, 28], and a theory of change has been elaborated on linking research on food safety in informal value chains to health outcomes [25].

## Conclusions

Reviewing food safety initiatives in Vietnam and elsewhere shows that improvements are possible, but are not always easy. Approaches that are based on working with the existing situation and gradually improving it have shown some success. However, these approaches cannot have long-term success unless they are accompanied by motivation for changing behaviour. For example, some of the new practices promoted such as less food spoilage will have obvious benefits, which can encourage adoption of these practices. In addition, new institutions can be introduced such as branding or licensing, which will act as an incentive for behaviour change for food safety actors as they have more incentive to change their current practices/behaviours. Where value chain stakeholders are not using modern food safety technologies, simple innovations such as food-grade containers or chlorinated water can result in substantial improvements to food safety and quality. Other technologies are effective and affordable but are not used; for example, adding lactoperoxidase to preserve milk or using chlorine washes to reduce bacteria on chicken carcasses. Risk analysis, Farm to Fork, and Hazard Analysis and Critical Control Points approaches have been very successful in improving food safety, but need adaption in order to apply them to the informal, wet markets in Vietnam, where most food is bought and sold.

Regulations are important, but regulations alone will never compel everyone to respect food safety. Nor can a food system transform overnight, and there are many aspects of smallholder production and traditional retail that are beneficial to Vietnam’s current stage of development. As such, improving current systems is advised, while also allowing development and modernisation. Finally, the Ministry of Health, Ministry of Agriculture and Rural Development, and Ministry of Industry and Trade – the three ministries responsible for food safety in Vietnam – should develop a better coordinated mechanism for food safety management among ministries and lower level of food safety authorities, such as at the province and district levels. It is important to continue developing a legislative framework, with a focus on simplicity, a clear mandate, flexibility and focus on food safety outcomes. In addition, the ministries and other related agencies should develop a coordinated plan for communicating in one voice with all affected parties during food safety crises so that the public and all related stakeholders can receive timely, clear and accurate information, which is informed by an understanding of human psychology, from credible sources to avoid unnecessary panic.

## Additional file

Additional file 1: Multilingual abstract in the five official working languages of the United Nations. (PDF 450 kb)

## Abbreviations

GAP: Good Agricultural Practices
ILRI: International Livestock Research Institute
VTV: Vietnam television
WHO: World Health Organization
