# Supplementary material for: Food safety in Vietnam: where we are at and what we can learn from international experiences
Source: Infect Dis Poverty. 2017 Feb 16;6:39. doi: 10.1186/s40249-017-0249-7 (PMC5314466; doi:10.1186/s40249-017-0249-7)
Supplement: Additional file 1: — Multilingual abstract in the five official working languages of the United Nations. (PDF 450 kb) [file 40249_2017_249_MOESM1_ESM.pdf]

Translation of the abstract into the five official working languages of the United Nations

سلامة الغذاء في فييتنام: أين نحن الآن وما يمكن أن نتعلمه من الخبرات الدولية

هونج نجوين فيت، تران ثي تويت هان، فريد أونجر، دانج زوان سين، ديليا جريس

ملخص
تجذب الأمراض المنقولة بالغذاء الكثير من الاهتمام في فييتنام كنتيجة لسلسلة مستمرة من الممارسات الغذائية الفاسدة وغير السليمة. وفي هذا البحث، سنقوم بتقديم بعض وجهات النظر حول سلامة الغذاء في فييتنام في إطار معهد بحثي دولي يعمل في مجال سلامة الغذاء بالتعاون مع شركاء له في البلاد. ونحن نسوق بأن أبرز المشكلات الخاصة بسلامة الغذاء في فييتنام تتلخص في أن بعض المستفيدين من سلسلة القيمة الغذائية ينقصهم أخلاقيات العمل، مما يؤدي إلى إنتاج وبيع أغذية غير سليمة من أجل تحقيق أرباح بغض النظر عن الآثار السلبية التي تمس صحة المستهلك. وفي المقابل، فإن عدم اتباع السلوكيات الأخلاقية فيما يتعلق بالغذاء يمكن أن يرجع إلى نقص العوامل المحفزة.
وبالرغم من أن سلامة الغذاء تتسبب في نشر حالة هلع بين المواطنين، إلا أنه إلى أي مدى تسهم الأغذية الملوثة في الأمراض المنقولة بالغذاء والتسمم الغذاء في فييتنام، فهذا الأمر ليس واضحًا. ومع ذلك، فإن المشكلات الصحية الأبرز على مستوى العالم والمرتبطة بالغذاء هي العدوى المتسببة من استهلاك أغذية ملوثة بالفيروسات أو البكتيريا أو الطفيليات. وتتمثل إحدى التحديات الخاصة بسلامة الغذاء في الطريقة غير المناسبة التي يتم من خلالها إعلان مخاطر الغذاء إلى العامة. كما أن هناك مشكلة أخرى رئيسية وهي الصعوبة المتاصلة في إدارة الأغذية في أسواق الأغذية وفي منافذ الانتاج الصغيرة. وعلى الصعيد الآخر، فتعد الأغذية المحلية والإنتاج والمعالجة الغذائية المحلية أحد الأركان الثقافية الهامة إلى جانب كونها جوهرية لسلامة الغذاء، وقد تتعرض تلك الجوانب إلى الخطر إذا أدت المخاوف المحاطة بسلامة الغذاء إلى تشجيع المستهلكين إلى شراء المزيد من الأغذية المستوردة. كما نناقش في هذا البحث الخبرات الجيدة في إدارة سلامة الغذاء في دول أخرى واستخلاص دروس مستفادة لفييتنام حول كيفية التعامل مع الوضع الحالي لسلامة الغذاء على نحوٍ أفضل.

Translated from English version into Arabic by Norhan Mahmo, through

TRANSLATORS
WITHOUT BORDERS

越南的食品安全：我们处于什么阶段以及可以从国际经验中学到什么？

Hung Nguyen-Viet, Tran Thi Tuyet Hanh, Fred Unger, Dang Xuan Sinh, Delia Grace

摘要
由于掺假和食品不安全行为时有发生，食源性疾病在越南正引起广泛关注。在国际研究机构与本国合作伙伴共同研究食品安全的背景下，本文提供关于越南食品安全的一些观点。我们认为，越南食品安全核心问题之一是由于某些食品价值链利益相关者缺乏道德，以致为获利去生产和交易不安全食品，而不顾对消费者的不利健康影响。
虽然食品安全可引起公众恐慌，但在越南受污染的食物导致食源性疾病和食物中毒的负担有多少还不清楚。然而，在全球，与食物相关的最大健康问题是食用受病毒、细菌或寄生虫污染的食物而引发的感染。食品安全的一个重要挑战是向公众不当地宣传食品风险问题。另一个关键的制约因素是管理农贸市场和小农户生产的食品所存在的困难。另一方面，当地食品

和当地食品的生产加工是重要的文化资产，对食品安全至关重要。如果由于对食品安全的关注刺激消费者购买更多的进口食品，那么这些方面可能面临风险。
在本文中，我们还讨论了其他国家食品安全管理好的经验，以及越南可以从中借鉴之处以更好地应对当前的食品安全形势。

Translated from English version into Chinese by Men-Bao Qian, through

CHINESE CENTER FOR DISEASE CONTROL AND PREVENTION
NATIONAL INSTITUTE OF PARASITIC DISEASES

Sécurité alimentaire au Vietnam : Où nous en sommes, et ce que nous pouvons apprendre des autres pays dans le monde

Hung Nguyen-Viet, Tran Thi Tuyet Hanh, Fred Unger, Dang Xuan Sinh, Delia Grace

RÉSUMÉ
Les maladies d'origine alimentaire sont dans la ligne de mire au Vietnam du fait de pratiques alimentaires dénaturées et dangereuses. Dans cet article, nous abordons le problème de la sécurité alimentaire au Vietnam, dans le contexte d'une institution de recherche internationale œuvrant à la sécurité alimentaire avec d'autres institutions et organismes dans le pays. Nous mettons en avant l'un des facteurs clés de la sécurité alimentaire in Vietnam, à savoir le manque d'éthique de certains acteurs dans la chaine de valeur de l'alimentation, ce qui entraîne la production et le commerce d'aliments potentiellement insalubres dans le seul but de faire des bénéfices, et sans prendre en compte les effets néfastes sur la santé des consommateurs. Par conséquent, le manque de pratiques déontologiques en ce qui concerne la nourriture peut être attribué à un manque de facteurs de motivation.
Bien que la question de la sécurité alimentaire fasse souffler un vent de panique sur la population, il est difficile d'évaluer précisément le degré d'incidence de la nourriture contaminée dans les cas recensés de maladies et d'intoxications d'origine alimentaire au Vietnam. Cependant, d'une manière générale, les infections sont la principale conséquence d'une consommation de nourriture contaminée par les virus, bactéries ou autres parasites. Le manque d'efficacité dans la communication des risques liés à la consommation d'aliments insalubres représente un problème majeur. Il est par ailleurs très difficile de contrôler les nourritures présentées sur les étals de marché au Vietnam par les petits agriculteurs et exploitants. Cela dit, les produits alimentaires locaux, ainsi que leur production et leur traitement, sont riches d'un point de vue culturel et essentiels en matière de sécurité alimentaire ; ces aspects peuvent être mis à mal si la question de la sécurité alimentaire incite les consommateurs à acheter davantage de denrées issues de l'importation.
Dans cet article, nous parlons également des expériences positives constatées dans d'autres pays en matière de gestion de la sécurité alimentaire, afin de s'en inspirer pour aider le Vietnam à mieux appréhender cette question essentielle.

Translated from English version into French by Word Pass, through

Пищевая безопасность во Вьетнаме: Текущее положение; что мы можем почерпнуть из опыта других стран

Hung Nguyen-Viet, Tran Thi Tuyet Hanh, Fred Unger, Dang Xuan Sinh, Delia Grace

АННОТАЦИЯ
Во Вьетнаме заболевания пищевого происхождения привлекают много внимания вследствие частых случаев употребления некачественных и опасных продуктов питания. В данной статье мы описываем точки зрения на безопасность продуктов питания во Вьетнаме в контексте работ в сфере управления безопасностью продуктов питания одного международного исследовательского института в сотрудничестве с партнёрами внутри страны. Мы считаем, что один из ключевых факторов, влияющих на безопасность продуктов питания во Вьетнаме, — непорядочность отдельных участников производственно-сбытовой цепи, которая приводит к производству и продаже ради прибыли вредных продуктов питания, независимо от их негативного влияния на здоровье потребителя. Неэтичное поведение в отношении продуктов питания можно, в свою очередь, отнести на отсутствие поощрительных мер или мотивирующих факторов.
Несмотря на то, что тревога о безопасности продуктов питания порождает панику среди населения, остаётся неясным, насколько заболеваемость болезнями пищевого происхождения и пищевые отравления во Вьетнаме порождены загрязнёнными продуктами питания. Однако в мировом масштабе крупнейшая медицинская проблема, связанная с пищей — это инфицирование из-за употребления пищи, загрязнённой бактериями, вирусами или паразитами. Крупной проблемой безопасности продуктов питания остаются неадекватные методы информирования общественности о рисках, связанных с продуктами питания. Другое ключевое препятствие состоит в неизбежных сложностях контроля продуктов питания на продуктовых рынках и от мелких фермеров. С другой стороны, местные продукты, местное производство и обработка продовольственных товаров — не только существенное условие пищевой безопасности, но и достояние культуры, и они могут быть подвергнуты риску, если потребители, руководствуясь соображениями пищевой безопасности, начнут покупать больше импортных продовольственных товаров.
В данной статье мы также рассмотрим удачный опыт управления безопасностью продуктов питания других стран, а также подведём итоги извлечённых Вьетнамом уроков о том, как успешнее справляться с текущей ситуацией.

Translated from English version into Russian by Elena Alieva, through

Seguridad alimentaria en Vietnam: Situación actual y qué podemos aprender de las experiencias internacionales

Hung Nguyen-Viet, Tran Thi Tuyet Hanh, Fred Unger, Dang Xuan Sinh, Delia Grace

RESUMEN

Las enfermedades transmitidas por alimentos actualmente atraen mucha atención en Vietnam como resultado de repetidos episodios de prácticas alimentarias riesgosas y con alimentos adulterados. En este artículo aportamos algunas perspectivas sobre seguridad alimentaria en Vietnam en el contexto de una institución de investigaciones internacionales que realiza trabajo sobre seguridad alimentaria con organismos asociados en el país. Sostenemos que uno de los problemas clave respecto a la seguridad alimentaria en Vietnam es que algunas de las partes interesadas en la cadena de valor alimentario carecen de ética, lo cual lleva a la producción y comercio de alimentos poco seguros con el fin de obtener ganancias, sin tomar en consideración los efectos adversos sobre la salud de los consumidores. A su vez, la insuficiencia de prácticas éticas en torno a los alimentos puede atribuirse a una carencia de incentivos o factores de motivación.

Aunque la seguridad alimentaria provoca pánico entre la población, no está claro cuántos alimentos contaminados contribuyen al lastre de las enfermedades transmitidas por alimentos en Vietnam. Sin embargo, globalmente el mayor problema de salud asociado con los alimentos son las infecciones adquiridas por el consumo de alimentos contaminados con virus, bacterias o parásitos. Un importante reto en cuestiones de seguridad alimentaria es la manera inapropiada en que se informa al público sobre riesgos alimentarios. Otra restricción clave es la dificultad inherente al manejo de alimentos en los mercados y la producción de minifundistas. Por otra parte, los alimentos locales y la producción y procesamiento locales de los alimentos tienen un importante valor cultural, además de ser esenciales para la seguridad alimentaria, y estos aspectos pueden ponerse en riesgo si las inquietudes sobre seguridad alimentaria motivan a los consumidores a adquirir más alimentos importados.

En este artículo, también hablamos sobre experiencias positivas en el manejo de seguridad alimentaria en otros países y aprovechamos las lecciones aprendidas para Vietnam sobre cómo manejar con mayor éxito la situación actual en materia de seguridad alimentaria.

Translated from English version into Spanish by Ana Tranorte, through
